# Next Generation Sequencing of Tracheal Aspirates in Children With Tracheostomy A Prospective Case‐Control Study

**DOI:** 10.1002/ppul.71511

**Published:** 2026-02-13

**Authors:** Pia Brensing, Julia Lutynski, Michael Hilder, Thorsten Brenner, Sonia Mazzitelli, Silke Grumaz, Florian Stehling

**Affiliations:** ^1^ Department of Pediatrics III, Pulmonology, University Hospital Essen University Duisburg‐Essen Essen North Rhine‐Westphalia Germany; ^2^ Department of Anesthesiology and Intensive Care Medicine, University Hospital Essen University Duisburg‐Essen Essen North Rhine‐Westphalia Germany; ^3^ Noscendo GmbH Duisburg North Rhine‐Westphalia Germany

**Keywords:** aspirates, children, colonization, NGS, tracheostomy

## Abstract

**Background:**

Tracheostomies are increasingly performed in children. The treatment of infections in tracheostomized patients is challenging because of the distinction between chronic colonization and acute infection. Next‐generation sequencing (NGS) is a promising alternative for identifying and quantifying pathogens in a short period. This study investigated the utility of NGS of tracheal aspirates from healthy and tracheostomized children (TC).

**Methods:**

This monocentric prospective case‐control study recruited children with long‐term TC and compared the colonization of the lower airways with a control group of children who had undergone elective general surgery. Tracheal aspirates were analyzed using three methods: bacterial culture, polymerase chain reaction (PCR), and NGS.

**Results:**

In total, 107 children were recruited for this study. Sixty‐two samples were excluded due to acute exacerbation, lack of tracheal secretion, or insufficient cfDNA concentration (< 0.3 ng/µl). Consequently, 45 samples (17 controls and 28 tracheotomized samples) were analyzed. Tracheal aspirates analyzed using NGS revealed a significantly higher prevalence of *Pseudomonas aeruginosa* (*p* = 0.0001), *Staphylococcus aureus* (*p* = 0.002), *Stenotrophomonas maltophilia* (*p* = 0.004), and *Moraxella catarrhalis* (*p* = 0.03) in TC. Through NGS, we were able to demonstrate that *Pseudomonas aeruginosa* was the dominant bacterium in 44% of the samples in which it was detected.

**Conclusions:**

NGS can effectively identify a wide spectrum of DNA‐based pathogens (bacteria, viruses, and fungi) in the tracheal aspirates of children. Tracheostomized children were significantly more likely to be colonized by difficult‐to‐treat bacteria. Future research is required to evaluate the clinical benefits of NGS compared with standard microbiological procedures.

**Abbreviation**
bp: base pairs
cfDNA: cell‐free Deoxyribo Nucleic Acid
CRP: c‐reactive protein
DSP: digital signal processor
IQR: interquartile range
NGS: Next‐Generation Sequencing
PCR: Polymerase Chain Reaction
QC: quality control
SD: standard deviation
TC: tracheostomized children

## 1 Introduction

Over the last decade, tracheostomies have been increasingly performed in children [1]. Pediatric tracheostomy is predominantly performed in children, who are generally categorized into one of two groups: (1) children with chronic respiratory insufficiency requiring prolonged ventilation due to cardiopulmonary or neurological diseases, and (2) children with airway obstruction due to craniofacial or airway anomalies mainly caused by laryngotracheal obstruction [2, 3]. Tracheostomized children (TC) have a high 5‐year mortality rate of up to 32% [4, 5]. Complications associated with tracheostomy include infection, hemorrhage, obstruction, displacement and granulation tissue development [2, 6]. Tracheostomy tubes are commonly colonized by multiple opportunistic pathogens, with *Pseudomonas aeruginosa* and *Staphylococcus aureus* being the most prevalent causative bacteria [1, 7, 8, 9]. Differentiating acute respiratory infections from chronic infections and infections from colonization can be challenging [3].

The individual microbiomes (colonization) of tracheostomized children are highly variable (displaying considerable beta diversity). Microbial alpha diversity describes the number of distinct taxa and the distribution of their relative abundances within a sample. A recent case‐control study revealed that tracheal aspirates from TC are characterized by reduced microbial alpha diversity and increased neutrophilic inflammation associated with oxidative stress response [4]. These findings underline the need for targeted therapeutic strategies to manage inflammation and maintain the microbial balance to reduce the risk of bacterial bronchopulmonary infections in the airways of TC, improve clinical outcomes, and reduce complications associated with long‐term tracheostomy [10].

The identification of infectious disease pathogens, particularly organisms with a low detection rate, is challenging and time‐consuming, even when traditional culture methods are used in combination with polymerase chain reaction (PCR) [11]. Consequently, differentiating between infection and colonization is often delayed or fails, resulting in longer hospitalization with high morbidity and mortality, particularly in critically ill children [12].

Next‐generation sequencing (NGS) can help identify causative microorganisms and thereby contribute to diagnostic and antimicrobial stewardship [11, 13]. NGS can detect pathogens with known DNA sequences in clinical samples, and is suitable for identifying rare and atypical microorganisms [14] by comparing specific nucleotide sequences to a large database containing the DNA sequences of microorganisms [15]. This high‐throughput sequencing method for diagnosing infectious diseases has been employed in several studies analyzing clinical blood samples [16, 17]. A recent meta‐analysis of adult patients with pulmonary infections revealed that NGS had a high diagnostic performance for pathogens in the bronchoalveolar lavage fluid, with a pooled sensitivity of 78% and specificity of 77% [18]. This method has the potential to become a routine diagnostic procedure owing to its higher sensitivity and faster turnaround time, given that the cost‐effectiveness improves [11, 18].

Although NGS has been successfully validated for use in bloodstream infections in adults, valid data for pediatric patients are pending [16]. One case series showed that NGS detected pathogens responsible for pediatric systemic inflammatory response syndrome rapidly and reliably, leading to therapeutic treatment changes [19]. However, to date, there are no data on the NGS analyses of tracheal aspirates in children. Therefore, the present study aimed to investigate whether NGS performed on children's tracheal aspirates can add valuable information to standard microbiology diagnostics (culture and PCR) and identify differences in colonization in pulmonary healthy and tracheostomized children.

## 2 Methods

### 2.1 Study Design

This monocentric prospective case‐control study recruited children from our pediatric department between April 2022 and May 2023. Children who underwent elective surgery for different reasons (predominantly orthopaedic or urogenital) in the absence of lung disease were included in the control group. In the tracheostomy group, tracheostomy had to be established at least 6 months prior to testing to avoid bias. All included children were free of acute respiratory tract infections based on their medical history, clinical examination, and laboratory findings (white blood cell count and C‐reactive protein [CRP] level). We aimed to assess the prevalence of bacterial colonization in the tracheal aspirates of children, as detected by NGS.

This study was approved by the Ethics Committee of the Medical Faculty at the University of Duisburg‐Essen (21‐10435‐BO). Written informed consent was obtained from the legal guardians of all children. Tracheal aspirates were collected following a standard operating procedure (two tracheal aspirates, each rinsed with 1 mL of NaCl 0.9% and labelled with the patient's pseudonym). Sample aliquots were shipped at ambient temperature, one to the in‐house microbiology department and one to Noscendo GmbH for NGS.

### 2.2 Standard Microbiology Procedures

Tracheal aspirate aliquots (10 µl) were inoculated in standard microbiological media (blood agar, chocolate agar, selective agar for gramnegative rods, chromogenic agar for yeasts and Malt extract agar for molds [200 µl each]). Depending on the medium, the incubation time varied between 48 h and 14 days (malt extract agar). Culture results were reported semi‐quantitatively. Species identification was conducted using a Vitek MS (bioMérieux, Nürtlingen, Germany). The automated Unyvero pneumonia multiplex HPN system (Curetis AG, Holzgerlingen, Germany) detects DNA of 21 bacterial species (Table S3), representing > 90% of etiological agents for bacterial pneumonia, and *Pneumocystis jirovecii* using 180 µl of the patient sample. The duration of the test was 4 h.

### 2.3 NGS

Tracheal aspirates were collected in stabilizing tubes (Cell‐Free DNA BCT® CE [Streck, La Vista, Nebraska, USA]), and shipped at ambient temperature to the collaborating laboratory of Noscendo GmbH in Tübingen, Germany, for sequencing and bioinformatic evaluation with their DISQVER® platform optimized for respiratory samples. For sequencing, tracheal aspirates were centrifuged at 16,000 rpm for 10 min at 4°C to remove any remaining cellular debris, after which supernatants were transferred to fresh reaction tubes. If necessary, volumes were adjusted to 1100 µl with 1 x phosphate buffered saline (Thermo Fisher Scientific, Waltham, Massachusetts, USA). DNA was isolated using a QIAsymphony DSP Circulating DNA Kit (Qiagen, Hilden, Germany) on a QIAsymphony DSP instrument (Qiagen). Cell‐Free DNA (cfDNA) concentrations were measured using a Qubit dsDNA high sensitivity assay (Thermo Fisher Scientific, Waltham, Massachusetts, USA), and samples with concentrations higher or equal to 0.32 ng/µl were used for library preparation on a Hamilton NGS STAR (Hamilton, Bonaduz, Switzerland) instrument. The sequencing libraries were quantified, and their quality was assessed using the Qubit kit and the Fragment Analyzer HS NGS Fragment assay (Agilent, Santa Clara, California, USA) on a Fragment Analyzer 5300 instrument (Agilent, Santa Clara, California, USA). Subsequently, samples that passed the quality assessment were sequenced with 75 base pairs (bp) read length and a minimum of 25 Mio reads per sample on an Illumina NextSeq. 1000 or NextSeq. 2000 instrument (Illumina, San Diego, California, USA).

The raw sequencing data were subjected to various quality control (QC) procedures, including Phred score filtering, adapter trimming, complexity filtering, k‐mer‐based classification, and contamination screening. To pass the quality filter, the read quality needed to surpass a Phred score of 20 and achieve a minimum length of 50 bp following QC. All generated data was analyzed using Noscendo's CE‐IVD platform DISQVER® with settings for respiratory samples. The results reported the read counts for each detected microorganism over DISQVER®platform within 24 h. Read count is the number of reads (short DNA sequence fragments) that could be mapped to a specific region in the genome. Therefore, they indicate the frequency at which DNA sequences from a sample are detected and mapped to a reference. NGS provides semiquantitative results.

### 2.4 Statistical Analysis

Statistical analyses were conducted using Excel 2016 and GraphPad Prism version 9. For all continuous variables with a normal distribution, we calculated the mean and standard deviation (SD), and the median was used with the interquartile range (IQR), if skewed. Discrete variables are presented as the counts and percentages. Missing values were not taken into account. Venn diagrams show the similarities and differences between the microbes detected using the three methods. We used the chi‐square test to compare two groups for significant differences (*p* < 0.05). The Spearman correlation test was conducted to show a monotonic relationship, where values of +1.0 show a perfect positive correlation. The Shannon test was applied to quantify the alpha diversity of detected microorganisms between samples. The higher the Shannon entropy, the greater the diversity.

## 3 Results

We recruited 107 children in our department, excluding 25 from the control group because of insufficient tracheal secretions present during surgery and 16 from the tracheotomized group due to acute exacerbation. For the NGS analysis, 21 samples (13 controls and 8 tracheotomized) were excluded as they did not pass the quality control criteria, defined as a minimum cfDNA amount of 0.3 ng/µl. Of the 21 samples that were excluded, the majority were negative on culture (13/21, 62%) or PCR (15/21, 71%) (data not shown). The overall positive agreement between the NGS results and the composite reference standard (culture and PCR) was calculated to be 72,6%. Consequently, 45 samples (17 controls and 28 tracheotomized) were eligible for the final analysis. The clinical and demographic data of the two groups are shown in Table 1. Children with tracheostomy were a median of 2 years older and were therefore taller and heavier. Males were predominant in both groups. Routine blood tests revealed no evidence of active infection. The underlying diseases that require tracheostomy are syndromic and neuromuscular diseases. The two main indications for elective intubation are urological and general pediatric surgeries.

**Table 1:** Baseline characteristics comparing the two groups.

variable	Control (*n* = 17)	Tracheostomized (*n* = 28)	Missing n Control/ tracheostomized
Age [years], median (IQR)	4 (3–7)	6 (2–10)	0/0
Female, *n* (%)	2 (12)	8 (29)	0/0
Height [cm], median (IQR)	103 (92–117)	114 (82–132)	1/2
Weight [kg], median (IQR)	16 (15–21)	19 (11–28)	0/0
Percentage premature < 38 weeks, *n* (%)	1 (6)	7 (25)	0/0
Leucocytes [per nL], mean ± SD	10 ± 4	10 ± 3	0/15
CRP [mg/dL], mean ± SD	0.4 ± 0	0.8 ± 0.9	0/18
years since tracheostomy, median (IQR)		4 (1–7)	‐/0
Main underlying diagnosis			
Lung disease, *n* (%)		2 (7)	‐/0
Central neurological disease, *n* (%)		6 (21)	
Neuromuscular disease, *n* (%)		8 (29)	
Syndromic disease, *n* (%)		12 (43)	
Ventilation modality			
Non, *n* (%)		5 (18)	‐/0
Intermittent, *n* (%)		13 (46)	
Continuously, *n* (%)		10 (36)	
Indication for elective intubation			
Urological surgery, *n* (%)	7 (41)		0/‐
General paediatric surgery, *n* (%)	9 (53)		
Imaging, *n* (%)	1 (6)		

Abbreviations: CRP, c‐reactive protein; IQR, interquartile range.

### 3.1 Diagnostic Yield of the Three Methods

**Figure 1:**
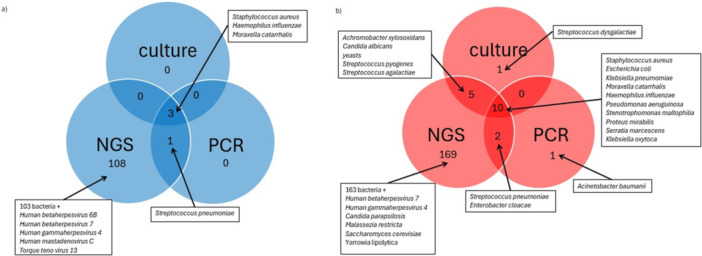
Venn diagram (a) control group (b) tracheotomized group. [Color figure can be viewed at wileyonlinelibrary.com]

First, the diagnostic yields of the three methods were compared. All methods detected more positive samples in the tracheotomized patients than in the healthy controls. In the NGS system, the normal oropharyngeal flora was not defined; therefore, tracheal colonization in healthy children was frequently reported to be positive (79%). The Venn diagrams of the two groups (Figure 1) describe the conforming and nonconforming findings of the different methods. The most frequent finding by NGS was bacteria; however, five viruses were detected in the control group, and two viruses and four fungi were detected in the tracheostomized group. *Candida parapsilosis* is a facultative pathogen, whereas the others could be considered transient flora. A list of all microorganisms detected by NGS is provided in Table S4. In the control group, all pathogens detected by culture or PCR were also detected using NGS. However, NGS detected 108 additional microorganisms (bacteria and viruses; Table S4). For the tracheotomized group, we identified more species using NGS than in the control group (186 vs. 112). The overlap between the employed methods was higher in TC, with the highest overlap being between NGS and culture (94%, 15/16), followed by NGS and PCR (92%, 12/13). Using NGS, 169 species that were not reported by culture or PCR were detected in TC.

### 3.2 Microbiome Differences Between Healthy and Tracheostomized Children

**Figure 2:**
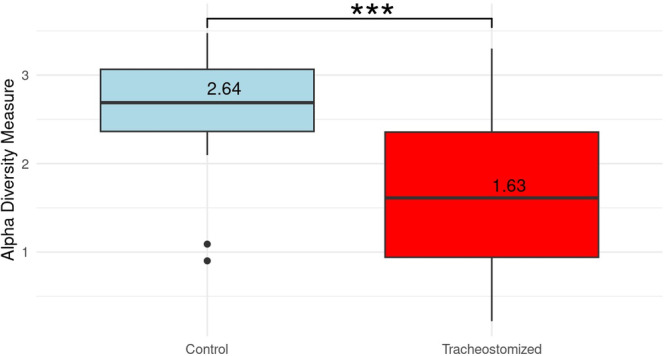
Shannon Test, performed over the total amount of species detected in control versus tracheotomized group, shows a significant reduction in alpha diversity in the tracheostomized group (p*** < 0.001). [Color figure can be viewed at wileyonlinelibrary.com]

In the subsequent step, we had a closer look at the differences between the two patient groups in the NGS analysis. The box plot in Figure 2 represents the alpha diversity of the microorganism spectrum comparing the two groups and highlights the significant differences in mean values and their distribution using the Shannon test (Figure 2, p***< 0.001). Higher Shannon values correspond to greater alpha diversity, reflecting higher taxonomic richness and more even relative abundance.

**Figure 3:**
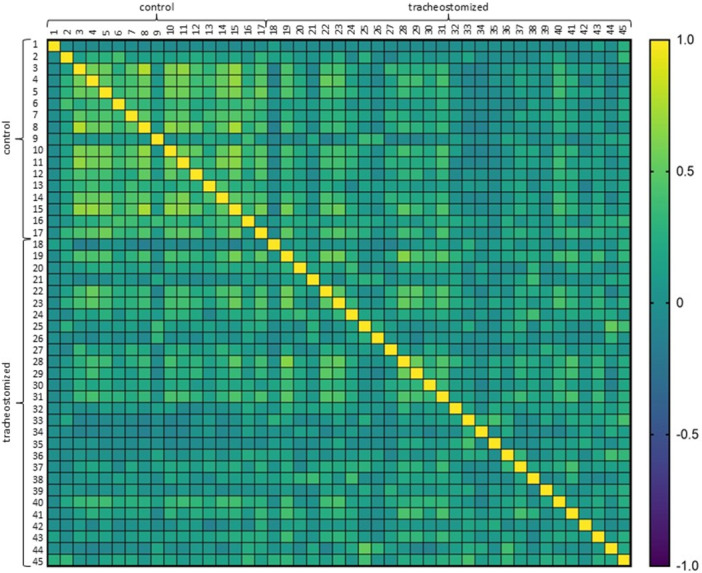
The heatmap depicts the Spearman correlation coefficients between control and tracheostomized groups. The colors represent the correlation (yellow positive, green no and purple negative correlation). [Color figure can be viewed at wileyonlinelibrary.com]

To investigate the possible correlation between the microbial profiles detected by NGS in healthy and tracheostomized children, the Spearman correlation test was applied, with results presented as a heatmap (Figure 3). The more similar the microbial profiles, the higher the correlation. According to the color classification, the heatmap indicated a positive correlation between control samples within the control group (mean Spearman correlation coefficient *r* = 0.36, Figure 3, upper left corner), whereas the samples from the tracheostomized group compared within this group indicated no correlation (mean Spearman correlation coefficient *r* = 0.17; Figure 3, lower right corner). The heatmap showed that the same species were identified with comparable abundance in the control group, whereas the species composition varied inter‐individually in the tracheostomized group.

**Figure 4:**
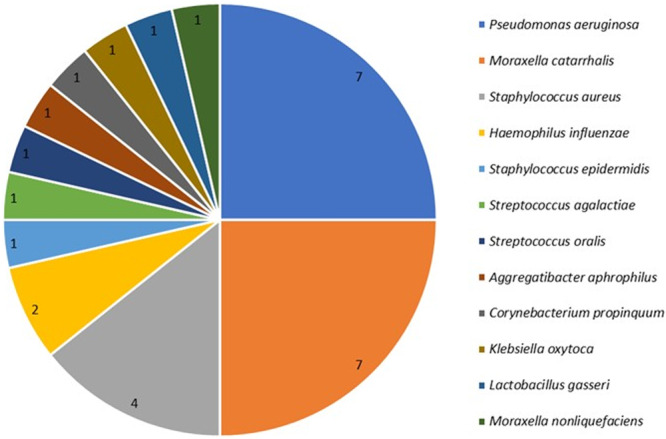
The dominant germ (highest read count in NGS) in every tracheostomized child (*n* = 28). [Color figure can be viewed at wileyonlinelibrary.com]

Colonization by specific bacteria, as detected using NGS, showed a significant difference between the two groups. When comparing the most common bacteria responsible for respiratory infections between the two groups, we found significantly more *Pseudomonas aeruginosa, Staphylococcus aureus, Stenotrophomonas maltophilia*, and *Moraxella catarrhalis* in the TC group (Table 2). Indeed, *Pseudomonas aeruginosa, Proteus mirabilis*, and *Enterobacter cloacae* were found only in children with tracheostomas (Table 2). Most TC were colonized by *Pseudomonas aeruginosa, Moraxella catarrhalis*, and *Staphylococcus aureus* (Table 2). The most abundant bacteria detected by NGS (highest read count of the sample) in the tracheostomized group were *Pseudomonas aeruginosa* and *Moraxella catarrhalis* (respectively 25%), followed by *Staphylococcus aureus* (14%) and *Haemophilus influenzae* (7%) (Figure 4). This overlap in prevalence is presented in Table 2.

**Table 2:** Results from NGS testing for the prevalence of colonization with the most common bacteria for respiratory infection compared to the two groups.

Bacteria	Control (*n* = 17)	Tracheostomized (*n* = 28)	*p* value
*Enterobacter cloacae*, *n* (%)	0	3 (11)	0.1624
*Haemophilus influenzae*, *n* (%)	4 (24)	12 (43)	0.1891
*Klebsiella pneumoniae*, *n* (%)	0	3 (11)	0.1624
*Moraxella catarrhalis*, *n* (%)	3 (18)	14 (50)	0.0300*
*Proteus mirabilis*, *n* (%)	0	5 (18)	0.0646
*Pseudomonas aeruginosa*, *n* (%)	0	16 (57)	0.0001**
*Staphylococcus aureus*, *n* (%)	1 (6)	14 (50)	0.0023**
*Stenotrophomonas maltophilia*, *n* (%)	1 (6)	13 (46)	0.0044**

*Note: p*‐values were calculated by chi‐square analysis and significance is indicated by *(*p* < 0.05) and **(*p* < 0.01).

## 4 Discussion

Overall, the present study aimed to investigate the diagnostic value of an NGS‐based microbiological work‐up of tracheal aspirates in children with chronic tracheostomy, as well as the prevalence of microbial colonization. This study detected through NGS a significantly higher frequency of colonization by *Pseudomonas aeruginosa*, *Staphylococcus aureus*, *Stenotrophomonas maltophilia*, and *Moraxella catarrhalis* in tracheostomized children than in pulmonary healthy children (Table 2). This is in line with several prior studies that detected 90–98% colonization with potential pathogenic bacteria in cultures of tracheal aspirates in tracheotomized children in the absence of an acute respiratory infection [7, 20]. Among these pathogens, *Pseudomonas aeruginosa* (56–67%), *Staphylococcus aureus* (9–77%), and *Stenotrophomonas maltophilia* (7 – 12%) have been identified as the most prevalent bacteria [7, 8, 20]. Using NGS, we found a comparable prevalence of *Pseudomonas aeruginosa* (57%) and *Staphylococcus aureus* (50%), and an even higher prevalence of colonization by *Stenotrophomonas maltophilia* (46%) in TC (Table 2). *Stenotrophomonas maltophilia* was detected in 19% and 37% of the TC group by culture and PCR, respectively (Supporting Figure 5). This discrepancy may reflect challenges in cultivating this bacterium. *Stenotrophomonas maltophilia* commonly colonizes patients with indwelling airway devices, and is frequently detected during or after antibiotic therapy, particularly with carbapenem antibiotics [21]. This could potentially be related to a higher, although undocumented, use of antibiotics in TC, which, in turn, may contribute to reduced microbial alpha diversity.

In addition, this study confirmed a significant reduction in alpha diversity in tracheotomized children (Figure 2), which is in line with a previous case‐control study of 33 tracheotomized children by Powell and colleagues [4]. Interestingly, reduced alpha diversity did not lead to a higher similarity within the tracheostomy group (beta diversity), as indicated by the lower Spearman correlation (Figure 3). This suggests that increased bronchopulmonary secretion associated with tracheostomy may lead to the overgrowth of a few inter‐individual different microorganisms. Consequently, there is an urgent need for subsequent clinical trials to perform subgroup analyses to identify specific species patterns of interindividual colonization in TC.

In situations of polymicrobial colonization, the causative microorganism of acute pulmonary exacerbations may not be defined. Through NGS, we were able to describe the mixed tracheal flora in TC, and demonstrate the dominant genus corresponding to the highest read counts. In TC, the highest read counts were observed for *Pseudomonas aeruginosa, Moraxella catarrhalis* and *Staphylococcus aureus* (Figure 4), which together accounted for two‐thirds (64%) of the patients´ dominant microorganism. The other way around, *Pseudomonas aeruginosa* was just in 7/16 positive NGS detections for *Pseudomonas aeruginosa* the dominant microorganism (44%). This is of particular importance as anti‐pseudomonal reserve antibiotic therapy may not be necessary in situations where *Pseudomonas aeruginosa* is not the dominant pathogen. *Pseudomonas aeruginosa* is frequently resistant to gentamycin, amikacin, and cefepime [20] and therefore, the current recommendations include piperacillin‐tazobactam for children with tracheostomy and acute lower respiratory tract infection [22]. Tracheostomy is a risk factor for colonization and infection with challenging to treat bacteria due to their common resistance to multiple antibiotics like *Pseudomonas aeruginosa, Staphylococcus aureus, Klebsiella pneumoniae, Acinetobacter baumannii, Enterobacter* species, and *Stenotrophomonas maltophilia* [20]. NGS may help to rapidly detect the dominant pathogen and, therefore, the most likely pathogen microorganism in acute respiratory tract infections.

In the present study, we only included children without respiratory tract infections, and the proportion of bacteria may change during pulmonary exacerbation. The implications of colonization during the infection‐free interval on the choice of antibiotic treatment during infection were not the target of this study. Nevertheless, our results are relevant for further investigations of whether NGS can help reduce the use of broad‐spectrum and reserve antibiotics in the treatment of acute exacerbations in tracheotomized children. However, NGS does not provide information on resistance and is relatively expensive.

The present study shows that NGS is a useful diagnostic tool for detecting a wide variety of microbes in the tracheal aspirates of children, expanding the spectrum of conventional diagnostics. The low percentage of negative predictive value of 27,4% is due to the high rate of positive NGS results in cases of negative cultures. This is a consequence of the fact that NGS reports every microorganism of the oropharyngeal flora as a positive signal where cultures from the microbiology department provide negative results, and summarize the bacteria as “normal flora” (Figure 1). Currently, an international definition of microorganisms, grouped as normal oropharyngeal flora, is lacking. Based on our small control group, it was not possible to identify the threshold of detection required to discriminate a pathogenic signal from a normal floral signal using NGS. Therefore, NGS results must be interpreted with caution and set in the clinical context to distinguish between relevant bacteria and oropharyngeal flora. It remains unclear if the high number of dropouts from NGS testing due to cfDNA < 0.3 ng/µl results from sterile samples. Supporting this assumption is the fact that most of the excluded samples were extracted from the control group (13/21, 62%), and approximately two‐thirds of the dropouts were negative by culture and PCR. Furthermore, it has to be taken into account that the used PCR‐testing was validated to detect pathogens in patients with clinical pneumonia, therefore the pathogen concentration has to reach around 10^2^ ^−3^ microorganisms/mL to generate a positive result. This study may serve as a basis for power analysis in larger studies to validate our results, analyze NGS in TC patients with acute respiratory infection, and establish standardized thresholds for NGS of tracheal aspirates.

### 4.1 Limitations

One limitation of this prospective study was its monocentric design, including only a small number of cases, with an unbalanced group size. However, we analyzed tracheotomized children, who are extremely difficult to recruit because of their rarity. In our healthy control group, it was difficult to collect tracheal aspirates that required general anesthesia for sampling. The general lack of data from case‐control trials on TC underlines these challenges. Pre‐testing confounders, such as recent antibiotic use, history of aspiration, and duration from sample collection to dispatch and analysis were not collected. With our study design, we were not able to distinguish between colonization and transient flora. Until now, there have been no validated thresholds or standard procedures for the NGS analysis of children's tracheal aspirates.

## 5 Conclusion

This is a pioneering study on the use of NGS as a supplementary method to test for microorganisms in tracheal aspirates of children with tracheostomy. NGS is a valid methodology for identifying a wide range of DNA microorganisms (bacteria, viruses, and fungi) in tracheal aspirates. Our study showed that through NGS testing, a significantly higher prevalence of colonization by *Pseudomonas aeruginosa, Staphylococcus aureus, Stenotrophomonas maltophilia* and *Moraxella catarrhalis* could be detected in the tracheal aspirates of tracheostomized children than in healthy children. Through NGS, we were able to demonstrate the dominant bacterium of colonization corresponding to the highest read counts and showed that *Pseudomonas aeruginosa* was the dominant bacterium in only 44% of the samples in which it was detected. There is an urgent need for larger studies to confirm the use of NGS in children's tracheal aspirates and to evaluate its use in clinical exacerbations in tracheostomized children to reduce the amount of reserve antibiotic therapy.

## Author Contributions

Concept and design: Florian Stehling; data collection: Julia Lutynski and Michael Hilder. statistical analysis and visualization: Pia Brensing, Sonia Mazzitelli, and Julia Lutynski. manuscript preparation: Pia Brensing. manuscript editing and review: Florian Stehling, Silke Grumaz, and Thorsten Brenner.

## Ethics Statement

This study was approved by the Ethics Committee of the Medical Faculty at the University of Duisburg‐Essen (21‐10435‐BO). Written informed consent was obtained from the patients legal guardians.

## Conflicts of Interest

The coauthors, Dr. Silke Grumaz and Sonia Mazzitelli, are both employees of Noscendo GmbH. The other authors declare no conflicts of interest.

## Supporting information


**Supporting Figure 5:** Showing the prevalence of three common bacteria in tracheostomized children analyzed by three different methods. **Supplementary Table 3:** List of all DNA bacteria detected in the used PCR panel. **Supplementary Table 4:** List of all microorganisms found through our NGS testing in tracheal aspirates of children.

supmat.docx.

## Data Availability

The anonymized primary data are available upon reasonable request.
