# Supplementary material for: Next Generation Sequencing of Tracheal Aspirates in Children With Tracheostomy A Prospective Case‐Control Study
Source: Pediatr Pulmonol. 2026 Feb 13;61(2):e71511. doi: 10.1002/ppul.71511 (PMC12903189; doi:10.1002/ppul.71511)
Supplement: Supplementary file 1 — Supporting Figure 5: Showing the prevalence of three common bacteria in tracheostomized children analyzed by three different methods. Supplementary Table 3: List of all DNA bacteria detected in the used PCR panel. Supplementary Table 4: List of all microorganisms found through our NGS testing in tracheal aspirates of children. [file PPUL-61-0-s001.docx]

Supplements


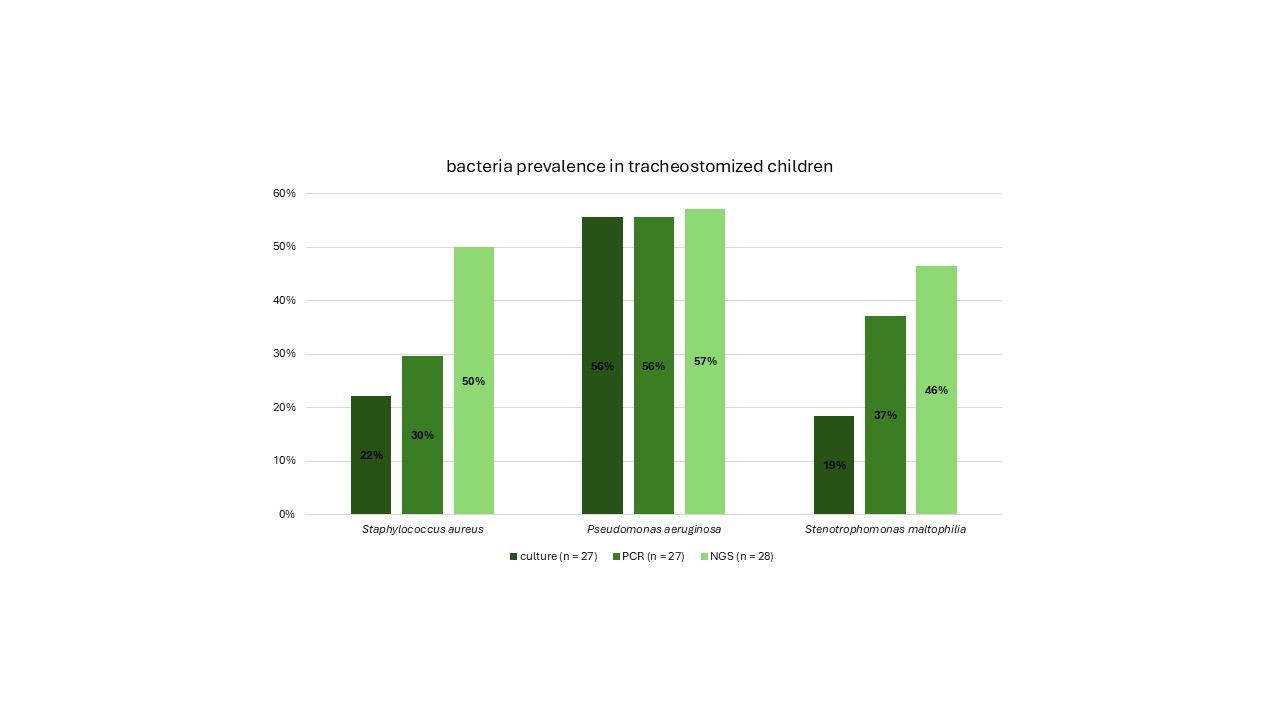


*Supplementary Figure 5: Showing the prevalence of three common bacteria in tracheostomized children analyzed by three different methods.*

*Supplementary Table 3: List of all DNA bacteria detected in the used PCR panel.*

| *Staphylococcus aureus* | *Proteus sp.* | *Klebsiella oxytoca* | *Moraxella catarrhalis* |
| --- | --- | --- | --- |
| *Streptococcus pneumoniae* | *Mycoplasma pneumoniae* | *Legionella pneumophila* | *Stenotrophomonas maltophilia* |
| *Escherichia coli* | *Serratia marcescens* | *Morganella morganii* | *Citrobacter freundii* |
| *Enterobacter cloacae complex* | *Haemophilus influenzae* | *Acinetobacter baumanii complex* | *Clamydophilia pneumoniae* |
| *Klebsiella aerogenes* | *Klebsiella pneumoniae* | *Klebsiella variicola* | *Pneumocystis jirovecii* |
| *Pseudomonas aeruginosa* |  |  |  |

*Supplementary Table 4: List of all microorganisms found through our NGS testing in tracheal aspirates of children*

| *Abiotrophia defectiva* | *Enterobacter cloacae* | *Neisseria elongata* | *Serratia marcescens* |
| --- | --- | --- | --- |
| *Achromobacter xylosoxidans* | *Enterococcus faecalis* | *Neisseria flavescens* | *Serratia spp.* |
| *Acinetobacter graevenitzii* | *Escherichia coli* | *Neisseria gonorrhoeae* | *Simonsiella muelleri* |
| *Acinetobacter Iwoffii* | *Filiafactor alocis* | *Neisseria lactamica* | *Slackia exigua* |
| *Acinetobacter junii* | *Finegoldia magna* | *Neisseria mucosa* | *Solobacterium moorei* |
| *Acinetobacter spp.* | *Fusobacerium hwasookii* | *Neisseria sicca* | *Sphingobacterium sp. B29* |
| *Acinetobacter pitii* | *Fusobacterium necrophorum* | *Neisseria spp.* | *Sphingobacterium sp. G1-14* |
| *Actinobacillus ureae* | *Fusobacterium nucleatum* | *Neisseria subflava* | *Sphingobium cloacae* |
| *Acinetobacter ursingii* | *Fusobacterium periodonticum* | *Niveispirillum cyanobacteriorum* | *Sphingobium sp. YG1* |
| *Actinomyces gerencseriae* | *Fusobacterium spp.* | *Novosphingobium pentaromativorans* | *Sphingobium yanoikuyae* |
| *Actinomyces graevenitzii* | *Gardnerella vaginalis* | *Novosphingobium resinovorum* | *Sphingopyxis lindanitolerans* |
| *Actinomyces massiliensis* | *Gemella haemolysans* | *Ochrobactrum anthropi* | *Sphingopyxis sp. 113P3* |
| *Actinomyces oris* | *Gemella morbillorum* | *Ochrobactrum intermedium* | *Sphingopyxis spp.* |
| *Actinomyces pacaensis* | *Gemella sanguinis* | *Oribacterium sinus* | *Staphylococcus aureus* |
| *Actinomyces spp.* | *Granulicatella adiacens* | *Ottowia sp. Oral taxon 894* | *Staphylococcus epidermidis* |
| *Aggregatibacter aphrophilus* | *Granulicatella elegans* | *Pannonibacter phragmitetus* | *Staphylococcus haemolyticus* |
| *Aggregatibacter actinomycetemcomitans* | *Haemophilus haemolyticus* | *Parvimonas micra* | *Staphylococcus hominis* |
| *Aggregatibacter segnis* | *Haemophilus influenzae* | *Pasteurella multocida* | *Stenotrophomonas maltophilia* |
| *Alcaligenes faecalis* | *Haemophilus parahaemolyticus* | *Peptoniphilus coxii* | *Streptococcus agalactiae* |
| *Angiostrongylus cantonensis* | *Haemophilus parainfluenzae* | *Peptoniphulus harei* | *Streptococcus anginosus* |
| *Bacteroides heparinolyticus* | *Haemophilus paraphrohaemolyticus* | *Peptoniphilus lacrimalis* | *Streptococcus cristatus* |
| *Bifidobacterium breve* | *Haemophilus sp. Oral taxon 036* | *Peptostreptococcus stomatis* | *Streptococcus constellatus* |
| *Bordetella spp.* | *Haemophilus spp.* | *Porphyrobacter sp. LM6* | *Streptococcus gordonii* |
| *Brevibacterium casei* | *Human betaherpesvirus 6B* | *Porphyromonas gingivalis* | *Streptococcus gwangjuense* |
| *Campylobacter concisus* | *Human betaherpesvirus 7* | *Prevotella bivia* | *Streptococcus infantis* |
| *Campylobacter showae* | *Human gammaherpesvirus 4* | *Prevotella buccalis* | *Streptococcus intermedius* |
| *Candidatus Saccharibacteria oral taxon TM7x* | *Human mastadenovirus C* | *Prevotella enoeca* | *Streptococcus milleri* |
| *Candida albicans* | *Kingella dentrificans* | *Prevotella intermedia* | *Streptococcus mitis* |
| *Candida orthopsilosis* | *Kingella oralis* | *Prevotella jejuni* | *Streptococcus mutans* |
| *Candida parapsilosis* | *Klebsiella oxytoca* | *Prevotella loescheii* | *Streptococcus oralis* |
| *Capnocytophaga gingivalis* | *Klebsiella pneumoniae* | *Prevotella melaninogenica 251* | *Streptococcus parasanguinis* |
| *Capnocytophaga granulosa* | *Klebsiella spp.* | *Prevotella nigrescens* | *Streptococcus pneumoniae* |
| *Capnocytophaga leadbetteri* | *Lachnoanaerobaculum umeaense* | *Prevotella oris* | *Streptococcus pseudopneumoniae* |
| *Capnocytophaga sputigena* | *Lactobacillus gasseri* | *Prevotella sp. Oral taxon 299* | *Streptococcus pyogenes* |
| *Capnocytophaga spp.* | *Lactobacillus johnsonii* | *Proteus mirabilis* | *Streptococcus salivarius* |
| *Cardiobacterium hominis* | *Lactobacillus spp.* | *Pseudoleptotrichia goodfellowii* | *Streptococcus sanguinis* |
| *Cardiobacterium valvarum* | *Lactococcus lactis* | *Pseudomonas aeruginosa* | *Streptococcus sp. A12* |
| *Chryseobacterium balustinum* | *Lautropia mirabilis* | *Pseudomonas putida* | *Streptococcus sp. NPS 308* |
| *Chyrseobacterium spp.* | *Leptotrichia shahii* | *Pseudomonas spp.* | *Streptococcus sp. Oral taxon 064* |
| *Corynebacterium accolens* | *Leptotrichia sp. Oral taxon 212* | *Pseudopropionibacterium propionicum* | *Streptococcus spp.* |
| *Corynebacterium jeikeium* | *Leptotrichia spp.* | *Ralstonia pickettii* | *Tannerella forsythia* |
| *Corynebacterium matruchotii* | *Limosilactobacillus fermentum* | *Rhizorhabdus dicambivorans* | *Tannerella sp. Oral taxon HOT-286* |
| *Corynebacterium propinquum* | *Malassezia restricta* | *Rothia aeria* | *Tardibacter chloracetimidivorans* |
| *Corynebacterium pseudodiphtheriticum* | *Megasphaera micronuciformis* | *Rothia dentocariosa* | *Torque teno virus 13* |
| *Corynebacterium spp.* | *Mogibacterium diversum* | *Rothia mucilaginosa* | *Treponema denticola* |
| *Corynebacterium striatum* | *Moraxella catarrhalis* | *Rothia spp.* | *Treponema ssp.* |
| *Cutibacterium acnes* | *Moraxella nonliquefaciens* | *Saccharomyces cerevisiae* | *Trueperella bernardiae* |
| *Dermabacter hominis* | *Moraxella osloensis* | *Schaalia odontolytica* | *Tsukamurella tyrosinosolvens* |
| *Dolosigranulum pigrum* | *Morganella morganii* | *Schaalia turicensis* | *Ureaplasma urealyticum* |
| *Edwardsiella anguillarum* | *Morococcus cerebrosus* | *Schistosoma japonicum* | *Veillonella dispar* |
| *Eikenella corrodens* | *Mycoplasma salivarium* | *Selenomonas sp. Oral taxon 126* | *Veillonella parvula* |
| *Elizabethkingia bruuniana* | *Neisseria bacilliformis* | *Selenomonas sputigena* | *Yarrowia lipolytica* |
| *Elizabethkingia miricola* | *Neisseria cinerea* | *Serratia liquefaciens* |  |
